# Impact of a School Mental Health Program on Children’s and Adolescents’ Socio-Emotional Skills and Psychosocial Difficulties

**DOI:** 10.3390/children9111661

**Published:** 2022-10-30

**Authors:** Aurora Adina Colomeischi, Diana Sinziana Duca, Liliana Bujor, Petruta Paraschiva Rusu, Ilaria Grazzani, Valeria Cavioni

**Affiliations:** 1 Faculty of Sciences of Education, Ștefan cel Mare, University of Suceava, 720229 Suceava, Romania; 2 “Riccardo Massa” Department of Human Sciences for Education, University of Milano-Bicocca, 20126 Milan, Italy

**Keywords:** mental health, social-emotional skills, risky behaviors, intervention effectiveness

## Abstract

The challenges of today’s society demand high levels of socio-emotional skills in children and adolescents; therefore, mental health is an important issue to be addressed and promoted in schools. The present study aims to investigate the effectiveness of a school mental health program (Promoting Mental Health at Schools; PROMEHS) designed to promote socio-emotional learning and prevent psychosocial difficulties in children and adolescents. The study was conducted on a sample of 1392 students (evaluated by 104 teachers) from kindergarten (*n* = 446), primary school (n = 426), secondary school (*n* = 354), and high school (*n* = 166). A quasi-experimental study design with experimental and waitlist control groups was used to evaluate the program’s effectiveness. Students were non-randomly assigned to the experimental (*n* = 895) and control group (*n* = 497). Students belonging to the experimental group received one-hour lessons once a week for 12 weeks. The teachers evaluated their students’ social-emotional skills, strengths, and difficulties before and after the intervention. The results indicated the effectiveness of the PROMEHS program in improving social-emotional skills for all school levels, reducing internalizing problems in primary and secondary school chil-dren, and reducing externalizing issues for kindergarten and primary school children. The PROMEHS program is a promising approach to enhancing childrens’ and adolescents’ social and emotional skills and to decreasing psychosocial difficulties, such as internalizing and externalizing problems.

## 1. Introduction

The World Health Organization defines mental health as a state of well-being in which the individual uses his skills, manages to cope with the stress of daily life, works productively and efficiently and can contribute to the community [1]. Research over the last decade has shown an increasing number of mental illnesses among young people, affecting between 10% and 20% of children and adolescents worldwide [2,3,4]. In addition, in the context of the COVID pandemic, the vulnerability of children and adolescents to develop mental disorders has increased due to social isolation and stress [5,6]. In this context, schools are one of the most suitable places to support the implementation of programs designed to promote and prevent mental health problems [7,8]. At the same time, in schools students are guided to establish their identity, build interpersonal relationships and other transferable skills such as socio-emotional skills, resilience, emotional intelligence, and behavioral regulation [9,10]. Also, schools are the settings where they are guided to reduce their internalized and externalized problems and antisocial behavior [11].

Mental health programs implemented in schools have compelling results that are mainly aimed at promoting Socio-Emotional Learning (SEL), resilience, the prevention of social, emotional and behavioral difficulties and the mitigation of risky behaviors [8,12,13]. SEL is defined as the process by which children, adolescents and adults are allowed to acquire and apply knowledge, skills and attitudes in order to develop a healthy identity, manage emotions and achieve personal and collective goals, feel and empathize with others, establish and maintain supportive relationships, and make responsible decisions [14,15]. In line with the principles of the Collaborative for Academic and Social and Emotional Education (CASEL) model, SEL contains five groups of basic social and emotional skills: self-awareness, self-management, social awareness, relationship skills, and responsible decision-making [16,17]. Two comprehensive meta-analyses that assessed the effects of implementing SEL intervention programs showed that students in schools where SEL curricula were applied showed improvements in socio-emotional skills, academic performance, self-confidence, positive attitude towards others and school, as well as a decrease of behavioral problems, emotional suffering and internalization symptoms [18,19]. Caldarella et al. [20] showed that second-grade students involved in a SEL program experienced an increase in prosocial behavior and a decrease in internalizing problems. Moreover, the SEL intervention had more substantial effects in the case of students at risk [20]. Another study found significant increases in prosocial behaviors of kindergarten children after applying a SEL curriculum [21]. In addition, a study testing the effectiveness of a socio-emotional learning program in Swedish schools showed significant effects in reducing children’s internalizing and externalizing problems and increasing self-management (self-efficacy and hopelessness), self-image, self-esteem and contentment [22].

Verissimo et al. [23] studied a group of 50 participants aged 10–13 who attended a high-risk public school and were exposed to environmental risk factors, such as domestic violence, poverty, and family dysfunction. They found significant changes in behavioral adjustment, anxiety, happiness and satisfaction, but the overall results showed that the intervention did not significantly impact emotional and behavioral problems [23]. In a sample of 1299 students, an intervention based on CASEL’s framework examined whether changes in self-awareness (i.e., emotional self-regulation, self-esteem, and self-reflection) were related to concurrent changes in youth’s positive development (i.e., resilience and psychological wellbeing) [24]. After 14 lessons implemented weekly for 90 min., the results indicated that increased emotional self-regulation and self-esteem were related to resilience and psychological wellbeing, but changes in self-reflection were not related to changes in resilience or psychological wellbeing [24].

Research shows that the impact of SEL programs might depend on the age of participants. There are studies showing that SEL programmes have the same effects in different age groups [25], while other studies found that interventions in childhood (ages 5–10) and early adolescence (ages 11–13) had the most significant follow-up effects compared to adolescents in high-school [26].

The present study aims to evaluate the Promoting Mental Health at School (PROMEHS) program’s effectiveness in Romanian students of various age groups. Seven European countries collaborated on this project: Italy, Romania, Portugal, Croatia, Greece, Latvia, and Malta. The objectives of PROMEHS were to design, implement, and evaluate a curriculum to promote mental health in schools for students, teachers, and parents. The curriculum aims to improve students’ learning and social and emotional resilience and reduce social, emotional, and behavioral difficulties [12,27]. A recent study evaluating the impact of the PROMEHS program across six European countries (Croatia, Greece, Italy, Latvia, Portugal, and Romania) showed that the PROMEHS program was effective in increasing social and emotional skills and prosocial behavior and decreasing mental health problems (i.e., externalizing and internalizing problems) [28].

The PROMEHS program includes the following components: (1) training and supervision courses for teachers; (2) activities with students using the handbooks designed for the program; and (3) meetings with school leaders and parents. In each participating country, a training support team was established to coordinate teacher training and the supervision, translation, and adaptation of textbooks and guides, as well as organizing and conducting meetings for school leaders and parents. The PROMEHS program development was guided by the following principles: whole school approach, SAFE (sequenced, active, focused, and explicit) approach [18], inclusive approach, providing quality training for teachers, and engaging the families. The PROMEHS program is designed to provide a systematic framework for the development and implementation of an evidence-based comprehensive universal mental health curriculum in schools. The PROMEHS curriculum was developed to address social and emotional learning, resilience, and emotional, social and behavioural difficulties. The program is focused on two dimensions: promotion (protective factors for mental health) and prevention. In order to promote social and emotional learning, the PROMEHS curriculum used the SEL model provided by CASEL [16,17], which is focused on self-awareness, self-management, social awareness, relationships skills, and responsible decision making. For resilience promotion, PROMEHS focuses on topics such as dealing with psychosocial challenges and dealing with traumatic experiences. The prevention dimension of the curriculum included the following topics: dealing with internalizing problems, externalizing problems, and risky behaviours. The activities include a brief description of students’ learning outcomes, targeted age, level of difficulty, and materials needed. Activities include storytelling, role plays, songs, videos, and games. The PROMEHS developers designed specific characters for the program (e.g., Ana, Liza, Sofia, Lucia, Jamal, John, Hong, Luca). The specific design of the activities includes: a short story about one or more PROMEHS characters, self-reflection questions, practical activities using different methodologies, evaluation chart to monitor students’ improvements, explanation of the aims of the activities, and instruction for teachers on how to embed the targeted skill into daily classes.

Considering the importance of cultural factors in predicting the efficacy of SEL programs and considering that the majority of SEL programs are implemented and tested in the US and Western Europe, the present study will describe the effects of the PROMEHS program on a rarely studied sample from Eastern Europe. Specifically, the present study will test the efficacy of PROMEHS in improving socio-emotional skills (i.e., self-awareness, self-management, social awareness, relationship skills, responsible decision making) and reducing psychosocial difficulties (i.e., internalizing and externalizing problems). The study also analyzed differences in the program efficacy according to the school level: kindergarten (4–5 years old children), primary school (8–9 years old children), lower secondary (11–12 years old children), and higher secondary (15–16 years old children). PROMEHS might be an important whole-school approach focused on socio-emotional skills and psychosocial difficulties, and is useful for teachers, school leaders, school counsellors, and parents.

Therefore, the following hypotheses were formulated:

**Hypothesis** **1 (H1).** *Students who attend the PROMEHS intervention will improve their socio-emotional skills (self-awareness, self-management, social awareness, relationship skills, responsible decision making) and will have lower levels of psychosocial difficulties (internalizing and externalizing problems) after completing the program compared with the situation before the intervention.*

**Hypothesis** **2 (H2).** *Students belonging to the experimental group will have higher scores in socio-emotional skills (self-awareness, self-management, social awareness, relationship skills, responsible decision making) and fewer psychosocial difficulties (internalizing and externalizing symptoms) compared with students in the control group. Further, we will explore the moderating role of school level in predicting the changes of socio-emotional skills and psychosocial difficulties.*

## 2. Materials and Methods

### 2.1. Research Context and Participants

This research is part of an international European project (ERASMUS +), Promoting Mental Health in Schools (PROMEHS), which aimed to develop a curriculum for promoting social-emotional learning and resilience, as well as for preventing behavioral problems in schools. Seven European countries collaborated on this project: Italy, Romania, Portugal, Croatia, Greece, Latvia, and Malta. Participating teachers were recruited by regional educational managers and school leaders. School managers and teachers agreed to participate in the study on a voluntary basis.

The data from the present study was collected from a sample of 104 teachers who evaluated 1392 students (724 females), 895 students in the experimental group and 497 in the control group. The sample size is sufficiently large to yield margins of error below 5%. According to the school level, 446 children attended kindergarten (4 to 5 years), 426 primary school (8 to 9 years), 354 lower secondary school (11 to 12 years), and 166 were high school students (15 to 16 years); a total of 9.1% of students were in a disadvantaged situation (students with special educational needs, Roma ethnicity, migrants). Students were matched by a code to combine pre-test and post-test scores. Regarding the sample of teachers, 61 teachers evaluated students from the experimental group and 43 teachers evaluated students from the control group. In terms of school level, 35 (19 experimental groups, 16 control groups) were from kindergarten, 27 (16 experimental groups, 11 control groups) from primary school, 26 teachers (13 experimental, 13 control) from secondary school, and 16 (13 experimental groups, 3 control group) from high school. The teachers evaluated their students’ social-emotional skills, strengths, and difficulties (Table 1).

### 2.2. Procedure

Data was collected in the first semester of the school year, in December 2020, and at the end of the school year, in June 2021. Participants were recruited through an information campaign run with the cooperation of the Suceava County School Inspectorate (representing the Romanian Ministry of Education). Schools agreed to participate in the study on a voluntary basis. Then, we sent information letters to participating schools for teachers and parents. The Institutional Review Board of the Romanian University approved the study. Teachers, parents, and students have been informed about the study and signed a consent form. Students were non-randomly assigned to one of the two groups. Students from the first schools volunteering to participate in the study were assigned to the experimental group and then a control group was established to meet the same criteria as the experimental group. Teachers completed a set of questionnaires before and after the intervention program. Teachers were instructed about the study ‘s purpose and the structure of questionnaires. Before completing the survey, all respondents gave their informed consent to participate. Students were identified by a code to combine the pre-test and post-test scores. Missing values within a scale were replaced by the mean of the scale. Then, nine subscales were generated by averaging their individual items’ rating scores. Internalizing difficulty, externalizing difficulty and total difficulty, self-awareness, self-management, social awareness, relationship skills, responsible decision-making, and total score SEL (social-emotional competence) were examined. A pilot study was conducted to assess the reliability of the questionnaires used for measuring the study variables.

The intervention was delivered to children by trained teachers. Teachers received 16 h of training focused on theoretical information and practical activities about mental health, social and emotional skills, resilience, and psychosocial issues in the school context. Teachers were also trained to use the PROMEHS handbooks developed for students (3–18 years), teachers, and parents. Students belonging to the experimental group received one-hour lessons once a week for 12 weeks as a part of the mainstream curriculum. During the implementation, teachers received 9 h of supervision.

### 2.3. Measures

The Strengths and Difficulties Questionnaire (SDQ) [29] is a 25-item questionnaire that measures psychosocial strengths and psychosocial difficulties of 3–16-year-old children and adolescents on a 3-point Likert scale ranging from 0 (not true) to 2 (certainly true). Previous research supported the factorial structure of SDQ, as well as validity on the Romanian population. A total difficulty score is computed by summing up the scores for the SDQ difficulty subscales. The present considered psychosocial difficulties (i.e., externalizing and internalizing) and total difficulties scores. The internal consistency of the scales is presented in Table 2.

The Social Skills Improvement System Social-Emotional Learning Brief Scales (SSIS SEL) [30] is a behavior rating form that allows teachers to evaluate the social and emotional skills of students as determined by the CASEL framework. In a recent study, the instrument was validated in the Romanian population [31]. The instrument consists of 5 scales that measure self-awareness, self-management, social awareness, relationship skills, and responsible decision-making. Items were rated on a 4-point Likert scale ranging from 0 (never) to 3 (almost always). For all scales, internal consistency coefficients were adequate (Table 2) [32].

### 2.4. Intervention

After the pre-test evaluation, teachers who applied the intervention for the experimental group received 16 h of training to implement the program. They applied the program’s activities in the classroom over 12 weeks. During this period, they received 9 h of mentoring and monitoring by qualified program trainers. The curriculum targeted three key themes for all school levels: (1) the promotion of social and emotional learning (SEL) (i.e., self-awareness, self-management, social awareness, relationship skills, responsible decision-making), (2) the promotion of resilience (i.e., dealing with psychosocial challenges and dealing with traumatic experiences), and (3) the prevention of social, emotional, and behavioral difficulties (i.e., externalizing difficulties, internalizing difficulties, and risk behaviors). Four PROMEHS handbooks have been developed for teachers and students for each school level (two handbooks for teachers and two handbooks for students). The handbooks addressed the same topics, but the activities have been designed specifically for pre-primary and primary school students (one handbook for teachers and one handbook for students with separate activities for kindergarten and for primary school students) and for lower secondary and higher secondary school children (one handbook for teachers and one handbook for students, including different activities for lower secondary and higher secondary students). The teachers’ handbooks include activities with the following structure: outcomes, targeted group, materials, activity steps, formative evaluation chart for teachers, suggestions on how teachers could embed the activity goals in other teaching subjects, and further resources. The student handbooks describe the following information for each activity: activity outcomes, materials, steps, and different resources such as stories, videos, exercises, worksheets, and suggestions of books or movies related to the topic. Furthermore, three guidelines have been developed for teachers, parents, and policymakers. The guideline for teachers discusses teachers’ mental health and presents different resources for teachers to prevent their own social, emotional, and behavioral problems and to promote resilience and positive emotions. The guideline for parents presents the principles and activities of the PROMEHS program, discusses the factors affecting mental health at home, and describes strategies for parents to promote socio-emotional learning and well-being. The guideline for policymakers discusses what social and emotional learning is, how they can be promoted, and offers recommendation for policy-makers in order to help them to develop policies for promoting mental health in schools. In addition, the PROMEHS program included two glossaries for teachers with definitions of concepts used in the handbooks. The handbooks, guidelines, and glossaries have been translated and adapted in the national languages of the seven countries involved in the project.

### 2.5. Design

A quasi-experimental study design with experimental and waitlist control groups was used to evaluate the program’s effectiveness [33,34]. Measures of dependent variables were obtained for each group before and after the introduction of the independent variable to the experimental group [34]. Students from the waiting list control group received PROMEHS training after finishing the intervention with students from the experimental group and after the post-test. In Romania, PROMEHS was implemented in 30 schools: 6 kindergartens, 14 primary and secondary schools, and 10 high schools.

### 2.6. Analytic Strategy

Differences between the experimental and control groups at pre-test were assessed using independent samples *t*-test. In order to test the two hypothesis (H1 and H2) and to analyze the efficacy of the program, we used two-way ANOVA for repeated measures, considering time of measurement (pre- and post-intervention) and group (experimental and control). For testing the moderating role of school level in predicting the changes of socio-emotional skills and psychosocial difficulties (H2), we used three-way ANOVA for exploring the interaction effects of school level (kindergarten, primary, lower secondary, higher secondary), group (experimental and control), and time of measurement (pre- and post-intervention). Considering that we had unequal sample sizes and unequal variances between samples, in order to conduct multiple comparisons for interaction effects, we applied the post hoc Bonferroni test [35]. All statistical analyzes were performed in Jamovi statistical software.

## 3. Results

### 3.1. Preliminary Analysis

First, a comparative analysis of the two groups was performed to check the homogeneity of the groups before intervention. The results of the independent samples *t*-test showed that for Internalizing (t_(1390)_ = 2.82, *p* = 0.005), Externalizing (t_(1390)_ = 2.05, *p* = 0.040), and total difficulties (SDQ)(t_(1390)_ = 2.74, *p* = 0.006), participants from the experimental group had significantly higher scores compared with those in the control group. Furthermore, students in the experimental group had significantly lower self-management scores than students in the control group (t_(1390)_ = −2.41, *p* = 0.016). Therefore, students from the experimental group had higher levels of psychosocial difficulties pre-intervention (such as internalizing and externalizing problems) and lower levels of socio-emotional skills in the domain of the self-management component. The groups were homogeneous in the other dimensions before the intervention (Table 3).

### 3.2. Effects of the Program

ANOVA for repeated measures was applied to test the two hypotheses. The scores of participants from both groups (experimental and control) were compared before and after the intervention. As can be seen in Table 4, the interaction between phase (pre- and post-intervention) and group (experimental and control) is significant for all dependent variables (p ≤ 0.05). The results showed that the “phase” factor has significant main effects in the case of all analyzed variables (p < 0.001), but the “group” factor has a significant main effect only for Self-awareness (p = 0.003) (Table 4).

To analyze the differences between the pre- and post-intervention phase (H1), but also to identify if there are differences between the experimental and control group (H2), the post hoc Bonferroni test was applied. The results showed significant increases in SEL variables, for the experimental group, at the post-intervention evaluation compared to the pre-intervention evaluation: self-awareness (t = −17.44, *p* < 0.001, η^2^ = 0.031), self-management (t = −13.47, *p* < 0.001, η^2^ = 0.005), social awareness (t = −13.56, *p* < 0.001, η^2^ = 0.019), relationship skills (t = 13.47, *p* < 0.001, η^2^ = 0.014), responsible decision-making (t = −11.17, *p* < 0.001, η^2^ = 0.013), total SEL (t = −16.22, *p* < 0.001, η^2^ = 0.027). The effect sizes are small (η^2^).

For Internalizing (t = 6.13, *p* < 0.001), Externalizing (t = 6.78, *p* < 0.001) and Total Difficulty (t = 7.51, *p* < 0.001), we found that only participants in the experimental group showed a significant decrease in scores post-intervention compared to the pre-intervention and the effect size is small (η^2^ p). Therefore, our findings support H1. 

Regarding the comparison of the results of the two groups of participants, to test the second hypothesis, we found that the students who participated in the PROMEHS program had significantly higher scores after the intervention than those in the control group. This trend is visible for all SEL variables such as self-awareness (t = 5.70, *p* < 0.001), self-management (t = 3.04, *p* < 0.014), social awareness (t = 3.88, *p* < 0.001), relationship skills (t = 3.02, *p* = 0.015), responsible decision-making (t = 3.28, *p* < 0.006), and SEL (t = 4.12, *p* < 0.001). The results confirm the second hypothesis for the SEL variables.

#### Analysis of the Moderating Effect of School Level

ANOVA for repeated measures was applied to analyze whether the intervention differed depending on school level (kindergarten, primary, lower secondary, and higher secondary), group (experimental and control), and phase (pre- and post-intervention) The results showed significant interaction effects between the three variables (Phase*Group*School Level) for Self-awareness (F = 5.15, *p* = 0.002, η2p = 0.011), Social awareness (F = 4.11, *p* = 0.006, η2p = 0.009, Relationship skills (F = 5.11, *p* = 0.002, η2p = 0.011), and Responsible decision making (F = 8.35, *p* = < 0.001, η2p = 0.018) and SEL (F = 4.53, *p* = 0.004, η2p = 0.010). The effect size value is small in all cases (η2p) (Table 5). To explore the three-way interaction further, we used the post hoc Bonferroni *t*-test and analyzed differences by phase (pre- and post-intervention) and group (experimental and control) for each school level.

The results showed significant differences in terms of ***Internalizing, Externalizing and Total Difficulty*** by phase for primary school students belonging to experimental group. The scores were significantly lower in the post-intervention group, compared with the pre-intervention group in primary school students from the experimental group, for the variables Internalizing (t = 3.89, *p* < 0.050), Externalizing (t = 6.83, *p* < 0.001), and Total Difficulty (t = 6.28, *p* < 0.001). According to the group, we found that pre-intervention, high school students from the experimental group had significantly higher scores compared with those from the control group for Internalizing (t = 6.40, *p* < 0.001), Externalizing (t = 4.81, *p* < 0.001), and Total Difficulty (t = 6.31, *p* < 0.001). Furthermore, post-intervention, high school students from the experimental group had higher scores in Internalizing (t = 6.09, *p* < 0.001), Externalizing (t = 3.76, *p* < 0.050), and Total Difficulty (t = 5.43, *p* < 0.001), compared with high school students from the control group (Table 5).

For ***Self-awareness***, significant differences were found by phase (pre- and post-intervention) for the experimental group for kindergarten, primary school, and high school students. Thus, post-intervention, the scores were significantly higher for the experimental group of students from kindergarten (t = −4.59, *p* < 0.001), primary school (t = −7.58, *p* < 0.001), and high school (t = −6.05, *p* < 0.001), compared with the pre-intervention group. (Table 5).

In terms of ***Self-management***, our findings indicated significant differences by phase (pre- and post-intervention) for the experimental groups for all school levels. Thus, post-intervention, the results showed significantly higher scores for self-management for the experimental group of students from kindergarten (t = −6.01, *p* < 0.001), primary school (t = −9.26, *p* < 0.001), lower secondary school (t = −5.58, *p* < 0.001), and high school (t = −5.89, *p* < 0.001) compared with the pre-intervention phase. In addition, we found that kindergarten students belonging to the experimental group had higher scores in Self-management post-intervention compared with students belonging to the control group (t = 3.87, *p* < 0.050). (Table 5.).

In the case of ***Social awareness***, significant differences were found by phase (pre- and post-intervention) in the experimental groups of students from kindergarten (t = −7.48, *p* < 0.001), lower secondary (t = −4.95, *p* < 0.001), and high school (t = −8.22, *p* < 0.001). The results were significantly higher post-intervention compared to pre-intervention phase. In terms of group, we found significantly higher scores on social awareness post-intervention for kindergarten students belonging to experimental group (t = 4.90, *p* < 0.001), compared to students belonging to the control group (Table 5).

Regarding ***Relationship skills***, the results showed significant differences by phase (pre- and post-intervention) for students belonging to experimental groups for all school levels. Therefore, students belonging to the experimental group from kindergarten (t = −5.35, *p* < 0.001), primary school (t = −7.32, *p* < 0.001), secondary school (t = −6.85, *p* < 0.001), and high school (t = −8.59, *p* < 0.001) had significantly higher scores on Relationship skills in the post-intervention phase, compared with the pre-intervention phase (Table 5).

In terms of ***Responsible decision making***, we found significant differences by phase (pre- and post-intervention) for the experimental groups of students from kindergarten (t = −4.36, *p* < 0.001), primary school (t = −7.57, *p* < 0. 001), lower school (t = −4.23, *p* < 0.001), and high school (t = −6.80, *p* < 0.001). In all four categories of students, the results of the experimental group in the post-intervention phase were significantly higher compared with the pre-intervention phase. Furthermore, the control group of kindergarten students recorded significantly higher results post-intervention compared with the pre-intervention phase (t = −5.16, *p* < 0.001). Differences by group (experimental and control) were found post-intervention for kindergarten students (t = 3.66, *p* < 0.001) (Table 5).

The overall score for the *SEL* variable differed by phase (pre- and post-intervention) in the case of the experimental groups at the kindergarten level (t = −7.87, *p* < 0.001), primary school (t = −9.93, *p* < 0.001), lower school (t = −6.49, *p* < 0.001), and high school (t = −8.74, *p* < 0.001). Therefore, students from all school levels belonging to the experimental group showed significantly higher scores on the SEL variable post-intervention compared with the pre-intervention phase. Regarding the differences by group (experimental and control), we found that for kindergarten students, the experimental group had significantly higher results than the control group post-intervention (t = 4.61, *p* < 0.050) (Table 5).

## 4. Discussion

The central aim of the current study was to test the efficacy of PROMEHS in promoting socio-emotional skills (i.e., self-awareness, self-management, social awareness, relationship skills, responsible decision making) and reducing psychosocial difficulties (i.e., internalizing, and externalizing problems). The two dimensions, promotion and prevention, were operationalized in goals and activities provided to the students belonging to the experimental group from kindergarten to primary, secondary, and to high school. Participants belonging to the experimental group practiced for 12 weeks, and in the school context, activities that positively impacted their mental health, were compared with the pre-intervention and control groups. The overall results and the interaction effects between the phase and group showed us that the intervention had a significant and positive effect on promoting mental health by improving social and emotional skills (i.e., self-awareness, self-management, social awareness, relationship skills, responsible decision making) and reducing internalizing and externalizing difficulties. Our findings are consistent with the findings of meta-analyses on school-based universal SEL interventions [18,26] that showed significant benefits of SEL programs in increasing social and emotional skills in all demographic groups for children with diverse developmental levels, races, and socio-economic status. In the case of internalizing and externalizing difficulties, decreases were observed after the intervention for the experimental group. These findings are consistent with other studies showing that SEL programs strongly impact prosocial behavior and internalizing and externalizing problems [12,18]. In addition, our results are in line with studies providing evidence for the efficacy of SEL programs in different countries, such as the US [36,37], Italy [38], Portugal [39], and Turkey [40]. Furthermore, the efficacy of the PROMEHS program supplements existing studies on SEL programs in Romanian schools [41,42,43].

Analyzing the effect of school level, we found that after the intervention, internalizing, externalizing problems, and total difficulty decreased significantly in primary school students. Our results are in line with other studies that identified similar effects of SEL programs on internalizing and externalizing difficulties [22,23,44,45].

For the SEL variables of self-management, relational skills, and responsible decision-making, students belonging to experimental group from all school levels (kindergarten, primary school, lower-secondary and higher secondary school) had significant improvements in the post-intervention assessment compared to the pre-intervention. In terms of self-awareness, our results showed significant differences pre-post intervention for kindergarten, primary and high school students. Social awareness increased after the intervention for kindergarten, lower-secondary and higher secondary students. These results are consistent with previous studies showing a positive impact of SEL programs on socio-emotional skills [23]. Specifically, existing research showed the efficacy of SEL programs in enhancing SEL competencies for kindergarten children [46], primary school students [45] secondary school students [47], and high school students [26]. Furthermore, our findings are in line with studies showing positive effects of SEL programs on students’ self-management skills [48], well-being (positive attitude, prosocial behavior, and academic performance) [26], social awareness [23], social skills and empathy [49]. The results of the present study support the importance of addressing socio-emotional skills and psychosocial difficulties in school intervention programs with students. PROMEHS might be an important whole-school approach, useful for teachers, school leaders, school counsellors and parents.

In terms of group differences, kindergarten students from the experimental group improved significantly compared with those in the control group regarding self-management, social awareness, responsible decision-making, and total SEL. These findings are in line with other studies suggesting that kindergarten students benefit from SEL programs [19,21]. The findings showing the efficacy of the PROMEHS program for younger children are in line with the results of existing meta-analyses [26,50,51]. Another study conducted on Romanian preschool children showed the efficacy of a prevention program inn reducing internalizing and externalizing problems of high-risk preschool children [43]. The significant improvements registered by the experimental group, compared with control group, for kindergarten SEL competencies, have an important value considering that most of the existing studies on kindergarten students did not include a control group [46]. Effective SEL programs might reduce discipline problems in preschool children and promote school readiness. A study conducted in Romania indicated that preschool teachers need training and support from mental health professionals in order to understand and prevent emotional and behavioral difficulties of children [52].

The present research study had several limitations, such as the use of teachers’ report measures and the lack of follow-up evaluations. Future longitudinal studies are needed to investigate the PROMEHS program’s effectiveness in the long term. In addition, analyzing students’ behavior from different perspectives (teachers, parents, students themselves) would be necessary for the future considering that evaluations of the teachers involved in the program implementation can be subjective [53]. Moreover, further studies investigating the efficacy of PROMEHS program should consider moderator variables, such as gender, socio-economic status class climate and teacher characteristics.

In terms of external validity of our findings, the PROMEHS program might be difficult to be implemented in schools due to the necessity of teachers training and supervision. In addition, the whole-school approach of the program and the quality implementation requires the involvement of students, teachers, parents, and school leaders. In Romania, optional subjects are chosen by the schools (School Decided Curriculum) and should be approved by the Ministry of Education. In general, optional school subjects are extensions of the compulsory subjects (e.g., Mathematics, Science) focused on knowledge development and formal education and do not encourage students’ personal development and social and emotional learning. Curriculum developers need to consider optional school subjects focused on life skills that will increase both students ‘well-being and performance. In order to further integrate the PROMEHS program in the curriculum as an optional school subject, it is important to increase the mental health awareness of school leaders, policy makers, and parents. Ensuring that school managers and teachers have a good understanding of the factors affecting the mental health of children and the importance of socio-emotional skills is essential for building healthy and happy schools.

## 5. Conclusions

This study indicates that PROMEHS is a promising universal mental health program for all schooling levels in Romania. Our findings showed improvements in students’ social and emotional skills and prosocial behavior and decreases internalizing and externalizing behaviors. Our intervention results and data from the literature review converge to sustain the importance of SEL programs for the healthy development of children, from kindergarten to high school, also from a promoting and preventing perspective. The indicators of the quality that accompanied the implementation of PROMEHS (reliability, dosage, quality, responsiveness, and adaptation) and the positive results in SEL skills provide scientific arguments for implementing the PROMEHS curriculum in schools. In order to prioritize the promotion of mental health in school through the PROMEHS program, the active involvement of managers and policymakers is essential to support the training of teachers in applying PROMEHS and activating time and space resources in the curriculum activities.

## Figures and Tables

**Table 1:** Sample of students by gender, status, and school level.

		Group		
		Experimental	Control	Pre-Post Phase
Gender	Male	440 (49.2%)	228 (45.9%)	668
	Female	455 (50.8%)	269 (54.1%)	724
Disadvantaged	Yes	86 (9.6%)	41 (8.2%)	127
	No	809 (90.4%)	456/91.8%)	1265
School Level	Kindergarten	246 (27.5%)	200 (40.2%)	446
	Primary	317 (35.4%)	109 (21.9%)	426
	Lower Secondary	224 (25%)	130 (26.2%)	354
	Higher Secondary	108 (12.1%)	58 (11.7%)	166
Total Sample Size		895 (100%)	497 (100%)	1392 (100%)

**Table 2:** Internal consistency of SDQ and SSIS-SEL in pre- and post-intervention.

		Cronbach’s Alpha	
	Scales	Pre-	Post-
SDQ	Internalizing	0.783	0.819
	Externalizing	0.851	0.849
	Total difficulty SDQ ^1^	0.733	0.780
SSIS SEL	Self-awareness	0.805	0.824
	Self-management	0.771	0.807
	Social awareness	0.847	0.867
	Relationship skills	0.826	0.855
	Responsible decision making	0.889	0.908
	Social emotional competence ^2^	0.954	0.963

^1^ Refers to composite scale combining the SDQ difficulty subscales. ^2^ Refers to composite scale combining the SSIS-SEL subscales.

**Table 3:** Independent samples *t*-test comparing pre-intervention scores for study variables between experimental and control group.

Dependent Variables	T	df	*P*
Internalizing	2.828	1390	0.005
Externalizing	2.055	1390	0.040
Total difficulty SDQ	2.742	1390	0.006
Self-awareness	−0.405	1390	0.685
Self-management	−2.410	1390	0.016
Social awareness	−0.822	1390	0.411
Relationship skills	−0.873	1390	0.383
Responsible decision making	−0.321	1390	0.748
Total SSIS SEL	−1.059	1390	0.290

**Table 4:** Mean, standard deviation, confidence interval, and significance of the main effects and interaction effects between phase and group.

Dependent Variables	Phase	Experimental Group		Control Group		Effect	F	Sig ^(3)^	η^2^ ^(4)^
		M(SD) ^(1)^	95% CI ^(2)^	M(SD) ^(1)^	95% CI ^(2)^				
Internalizing	Pre-	1.43 (0.32)	1.41–1.45	1.38 (0.31)	1.35–1.41	Phase	20.06	<0.001	0.014
						Group	2.75	0.097	0.002
	Post-	1.37 (0.34)	1.35–1.39	1.37 (0.33)	1.34–1.40	Phase X Group	8.15	0.004	0.006
Externalizing	Pre-	1.40 (0.37)	1.37–1.42	1.36 (0.38)	1.32–1.39	Phase	27.22	<0.001	0.019
	Post-	1.33 (0.38)	1.30–1.35	1.34 (0.39)	1.30–1.37	Group	0.885	0.347	0.001
						Phase X Group	8.43	0.004	0.006
Total difficulty	Pre-	1.41 (0.30)	1.39–1.44	1.37 (0.31)	1.33–1.37	Phase	31.8	<0.001	0.022
						Group	2.03	0.155	0.001
	Post-	1.35 (0.32)	1.34–1.39	1.35 (0.32)	1.32–1.38	Phase X Group	11.2	<0.001	0..008
Self-awareness	Pre-	2.95 (0.63)	2.91–2.99	2.97 (0.62)	2.91–3.02	Phase	199.9	<0.001	0.126
	Post-	3.29 (0.63)	3.25–3.33	3.09 (0.63)	3.03–3.14	Group	8.95	0.003	0.006
						Phase X Group	45.1	<0.001	0.031
Self-management	Pre-	3.04 (0.62)	3.00–3.09	3.13 (0.60)	3.07–3.18	Phase	99.9	<0.001	0.067
	Post-	3.29 (0.95)	3.25–3.33	3.19 (0.64)	3.13–3.24	Group	0.121	0.728	0.000
						Phase X Group	37.3	<0.001	0.026
Social awareness	Pre-	3.11 (0.65)	3.07–3.16	3.14 (0.65)	3.08–3.20	Phase	122.2	<.001	0.081
						Group	2.81	0.094	0.002
	Post-	3.38 (0.62)	3.34–3.42	3.24 (0.66)	3.18–3.29	Phase X Group	26.6	<0.001	0.019
Relationship skills	Pre-	3.13 (0.65)	3.08–3.17	3.16 (0.63)	3.10–3.21	Phase	136.6	<0.001	0.089
	Post-	3.38 (0.63)	3.34–3.42	3.27 (0.63)	3.22–3.33	Group	1.39	0.238	0.001
						Phase X Group	19.5	<0.001	0.014
Responsible decision making	Pre-	3.28 (0.66)	3.23–3.32	3.29 (0.68)	3.23–3.35	Phase	82.1	<.001	0.056
						Group	2.51	0.113	0.002
	Post-	3.48 (0.63)	3.44–3.52	3.36 (0.66)	3.30–3.42	Phase X Group	18.5	<0.001	0.013
SEL	Pre-	3.10 (0.57)	3.06–3.14	3.14 (0.58)	3.09–3.19	Phase	172.3	<0.001	0.110
						Group	2.87	0.091	0.002
	Post-	3.36 (0.57)	3.32–3.40	3,23 (0.60)	3.18–3.28	Phase X Group	39.2	<0.001	0.027

^(1)^ Mean (Standard deviation); ^(2)^ Confidence interval; ^(3)^ The significance of the effect; ^(4)^ Partial eta squared as measures of effect size.

**Table 5:** Mean, Post Hoc *t* Test, and significance of the interaction effect between phase, group, and school level.

	Gr	Phase	Mean					Post Hoc *t* Test by Phase					Post Hoc *t* Test by Group				Interaction Ph x Gr x SchL	
			K	P	LS	HS		K	P	LS	HS		K	P	LS	HS	F	η^2^
Int	E	preE	1.39	1.42	1.40	1.59	preE	1.99	3.89 *	3.30	3.24	preE	−1.11	0.55	1.84	6.40 **	1.00	0.002
		postE	1.36	1.36	1.34	1.50	postE					preC						
	C	preC	1.43	1.40	1.34	1.26	preC	0.38	0.81	1.77	2.44	postE	−1.96	−0.58	−0.60	6.09 *		
		postC	1.42	1.38	1.36	1.17	postC					postC						
Ext	E	preE	1.43	1.42	1.33	1.42	preE	2.82	6.83 **	0.91	2.31	preE	−1.22	0.96	1.81	4.81 **	1.37	0.003
		postE	1.38	1.30	1.31	1.35	postE					preC						
	C	preC	1.48	1.38	1.26	1.13	preC	1.95	0.63	−0.33	0.21	postE	−1.56	−1.44	1.10	3.76 *		
		postC	1.43	1.36	1.26	1.12	postC					postC						
TD	E	preE	1.41	1.42	1.37	1.51	preE	2.80	6.28 **	2.39	3.20	preE	−1.34	0.88	2.07	6.31 **	0.69	0.002
		postE	1.37	1.33	1.33	1.43	postE					preC						
	C	preC	1.45	1.39	1.30	1.19	preC	1.38	0.83	−0.69	1.49	postE	−1.96	−1.16	0.34	5.43 **		
		postC	1.43	1.37	1.31	1.14	postC					postC						
SeAw	E	preE	2.52	2.55	2.57	2.33	preE	−4.59 **	−7.58 **	−3.63	−6.05 **	preE	1.84	−0.89	−2.07	−5.17 **	5.15 *	0.011
		postE	2.63	2.72	2.67	2.56	postE					preC						
	C	preC	2.44	2.59	2.67	2.68	preC	−3.85 *	−0.99	−0.86	1.12	postE	2.10	1.96	−0.66	−0.99		
		postC	2.55	2.63	2.70	2.62	postC					postC						
SeMg	E	preE	3.04	3.03	3.14	2.90	preE	−6.01 **	−9.26 **	−5.58 **	−5.89 **	preE	2.05	−2.47	−0.62	−7.11 **	1.60	0.003
		postE	3.25	3.32	3.34	3.21	postE					preC						
	C	preC	2.92	3.20	3.18	3.60	preC	−2.76	−0.39	−0.53	−0.70	postE	3.87 *	1.48	2.04	−4.43 *		
		postC	3.02	3.22	3.21	3.65	postC					postC						
SoAw	E	preE	3.10	3.21	3.12	2.83	preE	−7.48 **	−7.36	−4.95 **	−8.22 **	preE	3.10	0.60	−1.98	−7.66 **	4.11 *	0.009
		postE	3.38	3.45	3.31	3.28	postE					preC						
	C	preC	2.91	3.17	3.26	3.62	preC	−4.17 *	−1.19	−1.12	0.25	postE	4.90 **	3.10	−0.06	−3.07		
		postC	3.08	3.24	3.32	3.60	postC					postC						
RS	E	preE	3.18	3.20	3.05	2.93	preE	−5.35 **	−7.32 **	−6.85 **	−8.59 **	preE	3.46	0.24	−2.82	−5.94 **	5.11 *	0.011
		postE	3.37	3.43	3.31	3.39	postE					preC						
	C	preC	2.97	3.19	3.25	3.54	preC	−5.06	−3.58 *	−0.75	−1.48	postE	3.32	2.77	0.30	−2.53		
		postC	3.17	3.24	3.28	3.65	postC					postC						
RDM	E	preE	3.21	3.30	3.35	3.20	preE	−4.36 *	−7.57 **	−4.23 *	−6.80 **	preE	4.66 *	−1.92	−1.34	−6.05 **	8.35 **	0.018
		postE	3.36	3.53	3.50	3.55	postE					preC						
	C	preC	2.94	3.44	3..45	3.84	preC	−5.16 **	−0.22	0.13	0.91	postE	3.66 *	1.13	0.90	−2.14		
		postC	3.14	3.45	3.44	3.77	postC					postC						
SEL	E	preE	3.09	3.15	3.12	2.95	preE	−7.87 **	−9.93 **	−6.49 **	−8.74 **	preE	−5.92 **	−0.68	−1.98	−7.18 **	4.53 *	0.010
		postE	3.33	3.41	3.33	3.36	postE					preC						
	C	preC	2.90	3.19	3.24	3.61	preC	−3.59 *	−0.91	−1.36	−0.47	postE	4.61 **	2.86	0.87	−3.02		
		postC	3.08	3.23	3.27	3.64	postC					postC						

Int—Internalizing; Ext—Externalizing; TD—Total Difficulty; SeAw—Self-awareness; SeMg—Self-management; SoAw—Social awareness; RS—Relationship skills; RDM—Responsible decision making; SEL—total score SEL; Gr—group; E—experimental; C—control; Ph—phase; pre—pre-intervention; post—post-intervention; K—Kindergarten; P—Primary; LS—Lower Secondary; HS—Higher Secondary; Ph x Gr x SchL—Interaction effect between phase, group and school level; η^2^ p—Partial eta squared as measures of effect size; * *p* < 0.050; ** *p* < 0.001.

## Data Availability

Data supporting reported results can be requested from the corresponding author upon reasonable request.
